# Immune Checkpoint in Glioblastoma: Promising and Challenging

**DOI:** 10.3389/fphar.2017.00242

**Published:** 2017-05-09

**Authors:** Jing Huang, Fangkun Liu, Zhixiong Liu, Hui Tang, Haishan Wu, Qianni Gong, Jindong Chen

**Affiliations:** ^1^Department of Psychiatry, the Second Xiangya Hospital, Central South UniversityChangsha, China; ^2^Mental Health Institute of the Second Xiangya Hospital, Chinese National Clinical Research Center on Mental Disorders (Xiangya), Chinese National Technology Institute on Mental Disorders, Hunan Key Laboratory of Psychiatry and Mental Health, Central South UniversityChangsha, China; ^3^Department of Neurosurgery, Xiangya Hospital, Central South University (CSU)Changsha, China; ^4^Department of Minimally Invasive Surgery, the Second Xiangya Hospital, Central South UniversityChangsha, China

**Keywords:** Glioblastoma, immunotherapy, checkpoint, CTLA-4, PD-1, PD-L1

## Abstract

Glioblastoma (GBM) is a severe malignant brain cancer with poor overall survival. Conventional intervention remains dismal to prevent recurrence and deterioration of GBM cell. Recent years have witnessed exciting breakthroughs in novel immune strategies, especially checkpoint inhibitors, some of which have become adjuvant setting after standard of care in melanoma. Several clinical trials of checkpoint inhibitors are ongoing in glioblastoma and other brain carcinomas. Plus, synergistic combinations of checkpoint inhibitors with conventional therapy strategies—radiotherapy, temozolomide, bevacizumab, and corticosteroids are now being exploited and applied in clinical settings. This review highlights the recent developments of checkpoints in GBM immunotherapy to provide a brief and comprehensive review of current treatment options. Furthermore, we will discuss challenges remained, such as unique immune system of central nervous system (CNS), immune-related toxicities, synergies, and adverse interactions of combination therapies.

## Introduction

Glioblastoma (GBM), the most common primary brain cancer (Chinot et al., 2014; Gedeon et al., 2014), is associated with an extremely aggressive clinical course and poor prognosis, despite the advance in therapies including surgical resection, chemotherapy, and radiotherapy (Mao et al., 2012; Topalian et al., 2012; Suryadevara et al., 2015). Standard-of-care therapy includes aggressive resection and radiation as well as temozolomide (TMZ) for newly diagnosed glioblastoma (Mao et al., 2012; Suryadevara et al., 2015), but the median overall survival (OS) remains a dismal 15–17 months (Topalian et al., 2012). One of the challenges in the treatment of glioblastoma is its aggressive growth characteristic. Complete surgical resection of glioblastoma is impossible due to infiltrative growth (Claes et al., 2007), multiple lesions (Thomas et al., 2013), and microscopic spread (Huang et al., 2016). Consequently, there is a strong need for new and effective therapeutic approaches for this disease.

The immunotherapy with checkpoint inhibitors in some tumors showed remarkable success in recent years (Topalian et al., 2012; Wolchok et al., 2013; Motzer et al., 2015; Rizvi et al., 2015). Pembrolizumab and nivolumab were the first two checkpoint inhibitors that target programmed cell death protein 1 (PD-1) approved by Food and Drug Administration (FDA) for metastatic melanoma in 2014, which showed high response rate with favorable toxicity (Wolchok, 2015). Nivolumab was approved by FDA for non-small-cell lung cancer (NSCLC) in 2015 and for head and neck cancer in 2016 (Wolchok, 2015; Michot et al., 2016). With the introduction of checkpoint inhibitors in cancer immunotherapy, checkpoint regulators raised a new hope as an appealing strategy in glioblastoma. In 2014, the first large phase III trial to study the effectiveness and safety of nivolumab in glioblastoma patients was initiated (NCT02017717). Another large phase III trial of nivolumab in glioblastoma patients was started in 2016 (NCT02617589). Here we highlight the benefits and limitations of checkpoint modulators in glioblastoma immunotherapy and summarize the clinical developments of checkpoint and its combination with other strategies. Also, we will discuss the challenges remained, such as unique immune system of CNS, immune-related toxicities, synergies, and adverse interactions of combination therapies.

## Immune checkpoint inhibitors

Immune checkpoints are defined as activity modulation of T-cell by co-stimulatory and co-inhibitory molecules to achieve an optimal immune response (Driessens et al., 2009; Chen and Flies, 2013). Immunity homeostasis requires orchestrating the interaction and modulation of these immune checkpoint mediators to optimize normal and appropriate immune responses and avoid autoimmune disorders in normal tissues as well (Reinherz and Schlossman, 1980; Harshyne et al., 2015; Baruch et al., 2016; Hutchinson, 2016; Jiang et al., 2016). CD28, a type of co-stimulatory molecules which is expressed in 90% CD4+ T-cell and 50% CD8+ T-cell, binds to cofactor B7 and up-regulates the effector T-cell activation (Ardon et al., 2012; Topalian et al., 2012; Hamid et al., 2013; Asaoka et al., 2015; Sznol and Longo, 2015; Wolchok, 2015). Conversely, Cytotoxic T-lymphocyte-associated antigen-4 (CTLA-4), also known as CD152, is capable of competitively binding to B7 and blocking co-stimulatory signals. It was first described in 1987 as a new member of the immunoglobulin superfamily (Brunet et al., 1987). In contrast to CD28 which is expressed constitutively on T cell surface, CTLA-4 is found only in the activated conventional T cells (Tconv) and CD4+Foxp3+ regulatory T cells. It has a 10- to 20-fold higher affinity to B7.1 (CD80) and B7.2 (CD86) (Liu and Zeng, 2012) compared to CD28 with 35% homology between each other. So CTLA-4 can suppress antigen-specific T-cell activation by interrupting costimulatory signaling and functioning as an inhibition in the naïve and memory T cells activation. Other data also suggested that CTLA-4 attenuates B7.1 and B7.2 expression on antigen-presenting cells (APCs) (Topalian and Sharpe, 2014). In addition, immune reactivity can be down-regulated by CTLA-4 mediating helper T-cell (Th) activity reduction. CTLA-4 also augments myeloid-derived suppressor cells (Topalian and Sharpe, 2014). In the absence of CTLA-4, the initial CD4+ gives Th-2 + T cell differentiation priority as well as secretes higher level of IL-4, IL-5, and IL-10 (Linsley et al., 1994). Kuehn and his colleagues demonstrated that CTLA4 haploinsufficiency can lead to dysregulation of FoxP3+ regulatory T cells (Tregs) and is related to B cells reduction in lymphoid organs through the increase in autoreactive CD21 B cells (Lesterhuis et al., 2013; Kuehn et al., 2014).

Programmed death ligand 1 (PD-L1), a B7 homolog 1 (B7-H1) is expressed by tumor cells, APCs, B cells, and parenchymal cells (Dong et al., 1999). It can induce T cell apoptosis or anergy by binding to its receptor programmed death 1 (PD-1), which expresses on activated T cells mainly in peripheral organs and local sites of inflammation (Takita et al., 2006; Ansell et al., 2015; Baruch et al., 2016). It functions to suppress T-cell activation in a similar manner but distinct kinetics with CTLA-4 (Linsley et al., 1994). Besides, PD-1 is also involved in T-cell priming by dendritic cells (DCs) and promotes Tregs proliferation as well as B cell and natural killer (NK) cell responses reduction (Jackson et al., 2014; Taube et al., 2014; Gibney et al., 2015; Gryaznova et al., 2016). PD-L1 expression can be augmented by inflammatory cytokines, particularly interferons, and at the same time PD-L1 promotes tumor-specific interferon-γ production of CD8 + T cells (Ding et al., 2014; Wang et al., 2014). PD-L1 is expressed in multiple tumors including glioblastoma and melanoma (Dong et al., 2002; Zang and Allison, 2007). In GBM, expression of PD-L1 on the surface of tumor cells has been linked to the phosphatase and tensin homolog (PTEN) loss and PI3K-PTEN-AKT-mTOR signaling pathway overactivation (Mao et al., 2012; Topalian et al., 2012). Thus, multiple aspects of immune reactivity can be enhanced by therapeutic targeting of PD-1 associated with Tregs, cytotoxic T cells, B cells, and NK cells (Melero and Lasarte, 2015; De Vries and Figdor, 2016; Kranz et al., 2016). Lymphocyte-activation gene (LAG-3) and B- and T-lymphocyte attenuator (BTLA) are also among immune checkpoint inhibitors that can attenuate T-cell activation through diverse pathways (Durham et al., 2014; Baruch et al., 2016; Jiang et al., 2016).

Furthermore, there is interest in checkpoints expressed in other immune cell populations such as natural killer (NK) cells. Delconte et al. found that the suppressor of cytokine signaling (SOCS) family member cytokine-inducible SH2-containing protein (CIS) functions as a crucial intracellular negative regulator of activated NK cells. More importantly, they showed that CIS blockage increased the antitumor activity of NK cells (Delconte et al., 2016). The authors also found that combination of CIS inhibition with CTLA4 and PD1 blockade had a greater effect in reducing melanoma metastasis than either of these treatments alone. CIS inhibition may offer an alternative therapeutic option for patients who failed with other immune checkpoint inhibitors. The potential for NK-targeted agents to augment the antitumor effects of T cell checkpoint blockade is actively under consideration. A number of promising NK-targeting therapeutics are in early-phase trials.

Cancer cells can exploit immune checkpoints to evade immune attack and suppress immune destruction. CTLA-4 is overexpressed in activated CD4+T cell and CD8+T cell in tumor microenvironment (Brahmer and Pardoll, 2013). The expression of PD-1 by tumor-infiltrating lymphocytes accompanied with PD-L1 on tumor cells was detected by a variety of studies (Harshman et al., 2014). They are expressed excessively on the surface of cancer cells so as to silence T cell signaling and promote resistance in the tumor microenvironment (Wang et al., 2014). The mechanisms of overexpression include inactivating mutation of PTEN tumor suppressor (Lesterhuis et al., 2013; Naidoo et al., 2014) and secreting massive inflammatory cytokines by tumor cells, especially IFN-γ which induces PD-L1 expression (Naidoo et al., 2014; van Dam et al., 2014).

Preclinical trials as well as various stages of clinical trials have proved the efficacy and safety of several types of immune checkpoints inhibitors (Silk et al., 2013; Suryadevara et al., 2015; Hassel, 2016). In 1996, CTLA-4 inhibitors monoclonal antibody was firstly reported to lead to tumor regressions in murine model (Tang et al., 1996). And preclinical studies already proved that CTLA-4 inhibition achieved prolonged overall survival and stabilization on GL-261 tumor-bearing mice and immunocompetent VM/Dk mice, along with considerable safety and toleration. In addition, evidence on radiography and immunohistochemistry proved that mice survived for a long term demonstrated potent immunological memory (Wainwright et al., 2014). At the meantime, PD-1/PD-L1 inhibitors in preclinical data demonstrated that anti-PD-1 mAb combined with localized radiation can improve long-term survival modestly among C57BL/6 mice with GL-261 intracranial tumors (Zeng et al., 2013; Wainwright et al., 2014). A cohort of long-term survivors showed no sign of tumor growth, indicating the immunological memory establishment.

Because of the fact that CTLA-4 and PD-1 play a crucial regulatory role in tumor immunoreaction process, their inhibitors have been well studied for a long period and demonstrated exciting benefit in clinical cancer therapy. Ipilimumab, known as a fully humanized IgG1 subclass monoclonal antibody (mAb) against CTLA-4, demonstrated significant antitumor power while conventional therapies of metastatic melanoma remained dismal. It was approved for melanoma therapy by FDA in 2011 and became part of routine melanoma treatment paradigms (Danlos et al., 2015; 2016; Rosell and Karachaliou, 2016). It also improved immune related progression free survival greatly in non-small cell lung cancer (NSCLC) when combined with chemotherapy. Another humanized anti-CTLA-4 antibody, tremelimumab obtained durable responses in phase I/II clinical studies with melanoma but fell short in Phase III randomized clinical trial (Boussiotis, 2014; Topalian et al., 2014; Deng et al., 2015; Sznol and Longo, 2015; Kataoka et al., 2016).

The PD-1/PD-L1 axis has also shown to be a potential target in tumor tissues (Topalian et al., 2012; Dovedi et al., 2014; Derer et al., 2016). PD-1 inhibitor nivolumab demonstrated extended survival or maintenance of response in patients with advanced melanoma (Topalian et al., 2014). Objective responses produced by anti-PD-1 antibody were observed in ~20–25% patients with NSCLC, melanoma, or renal-cell cancer (Topalian et al., 2012; Taube et al., 2014; Wolchok, 2015). Besides, association between anticancer treatment response and pre-treatment tumor PD-L1 expression has been observed in early clinical trials (Harshman et al., 2014; Sharon et al., 2014). But initially it can only be achieved when combined with certain vaccine (Larkin et al., 2015). In September 2016, the United States granted anti-PD-1 drug, pembrolizumab as treatment in metastatic melanoma after standard of care treatment (Robert et al., 2015). Besides, Ansell et al. showed that nivolumab achieved considerably encouraging objective response rate (87%) in relapsed or refractory Hodgkin's lymphoma. Furthermore, therapeutic targeting of CTLA-4 or PD-1 was also proved to achieve durable tumor regression in NSCLC, bladder cancer, and renal cell carcinoma (Domingues et al., 2014; Kyi and Postow, 2014; Roth et al., 2016).

## Interaction between the immune system and GBM

The central nervous system (CNS) has been traditionally assumed as “immune privileged” organ. Intact blood-brain barrier (BBB), absence of conventional lymphatic system and low levels of APCs, MHC, and T cells limit the immune responses in the brain (Carson et al., 2006). This classical dogma that CNS is immune-privileged and lacks immuno-surveillance has been challenged by several studies, implying that the CNS interacts dynamically with the systemic immune system. In 2015, a CNS lymphatic system was discovered, in which the CNS antigens and T cells can reach the deep cervical lymph nodes through cerebrospinal fluid-filled channels (Louveau et al., 2015). Migrated APCs from the CNS present antigen to T-cells and can return to the CNS perivascular spaces. The disruption and increased permeability of BBB by injury, inflammation, and tumor also contribute to the interaction between the CNS and immune system. Furthermore, it is also clear that immune cells can enter the CNS in various neurological diseases (Cserr et al., 1992; Ohtsuki and Terasaki, 2007; Roopenian and Akilesh, 2007). Antigen-specific T cells response to CNS antigens in multiple sclerosis indicated that CNS is permissive for antigen-specific immunity from periphery. To confirm this communication, more studies indicated that a lymphatic system existed in which leukocytes can be shuttled by lymphatic vessels to CNS with an intact BBB (Claes et al., 2007; Thomas et al., 2013; Aspelund et al., 2015; Louveau et al., 2015). Taken together, CNS actively communicates with the immune system.

## Immune response and checkpoints in glioblastoma

**Figure 1:**
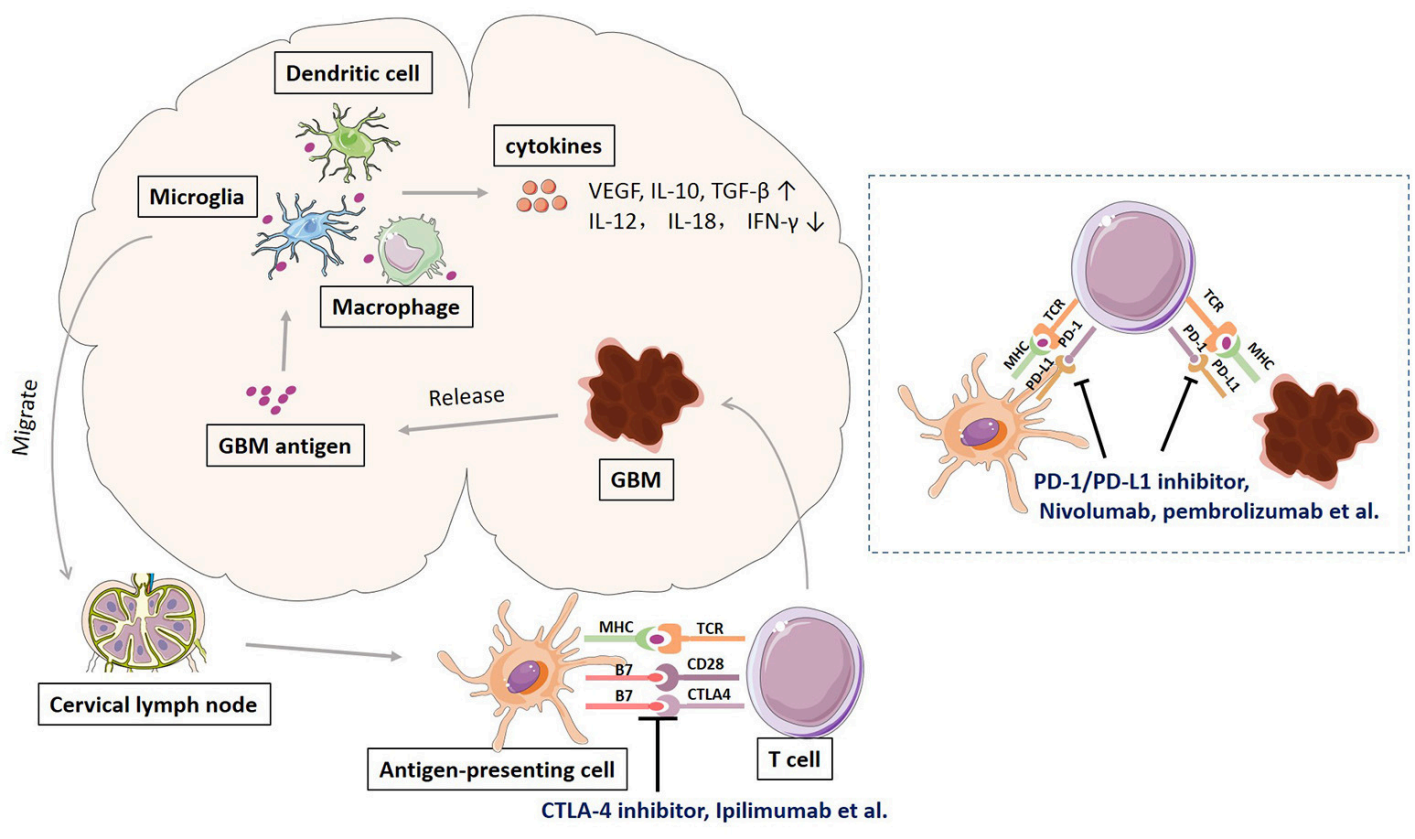
**Schematic representation of immune response and checkpoints in glioblastoma immunotherapy**.

Glioblastoma, like many other cancers, activates local immune response, and at the same time increases immune checkpoint protein expression to avoid immune attack. Schematic representation of immune response and checkpoints in glioblastoma immunotherapy was shown in Figure 1. The antigens released by glioblastoma tumor cells are taken by tumor-associated macrophages (TAMs), dentritic cells, and microglias. Microglia is the major innate immune cells in the CNS with critical functions. It responds quickly to pathogens and injuries also produces various cytokines (Graeber et al., 2002; Reardon et al., 2014). These APCs will release immunosuppressive and pro-tumorigenic cytokines into the GBM microenvironment. The production of immune inhibitory cytokines incudes transforming growth factor-b (TGF-β), vascular endothelial growth factor (VEGF), and interleukin-10 (IL-10) (Reardon et al., 2014). The disruption of BBB by GBM tumor cell invasion facilitates the drainage of APCs and CNS antigens to the peripheral lymph nodes (Graeber et al., 2002; Reardon et al., 2014). GBM antigens reach the peripheral lymph nodes via migration of APCs and drainage via lymphatic vessels. In the peripheral lymph tissues, the interaction between T cells and GBM is through antigen presentation to T cells and T cell priming. The interaction is regulated by multitude co-stimulatory (CD80, CD86, CD28) and co-inhibitory (CTLA-4) immune checkpoints molecules, which can be blocked by CTLA-4 inhibitors such as ipilimumab and tremelimumab (Reardon et al., 2014; Razavi et al., 2016). Activated T cells return the CNS and interact with tumor cells, which can be regulated by PD-1 and PD-L1 inhibition. PD-1 is induced and presents on activated T cell. PD-L1 expressed on GBM tumor cells and microglias binds to PD-1 to negatively regulate immune responses caused by T cells. PD-1 inhibitors (nivolumab, labrolizumab, pidilizumab) and PD-L1 inhibitors (BMS-936559, MPDL3280A, MEDI4736) block this immunosuppressive mechanism and increase GBM tumor cell destruction (Razavi et al., 2016).

Immune checkpoint inhibitors have demonstrated promising benefits for patients with GBM in several studies. In 2014, the first large phase III trial of nivolumab plus ipilimumab in recurrent glioblastoma (NCT02017717) was initiated. Besides, pidilizumab, as another PD-1 inhibitor is on clinical phase I and II testing the safety, toxicities, and efficacy in Relapsed GBM (NCT01952769). Representative clinical trials of checkpoint inhibitors in glioblastoma and brain metastases were summarized in Table 1.

**Table 1:** **Representative clinical trials of immune checkpoint inhibitors in glioblastoma and brain metastases**.

**Trial identifier**	**Eligible disease conditions**	**Targets**	**Treatments**	**Estimated enrollment**	**Start date**	**Clinical phase**	**Primary outcome**
NCT02617589	Glioblastoma	PD-1	Nivolumab, Temozolomide, Radiotherapy	*n* = 550	2016	III	Overall survival (OS)
NCT02667587	Glioblastoma	PD-1	Nivolumab, Temozolomide, Radiotherapy, Nivolumab placebo	*n* = 320	2016	II	Overall survival defined as time from the date of randomization to the date of death
NCT02529072	Recurrent brain tumors	PD-1	Nivolumab, DC vaccine	*n* = 66	2016	I	The safety of administering DC vaccines with nivolumab, as measured by the percentage of patients who experience unacceptable toxicity during combination treatment
NCT02648633	Recurrent glioblastoma	PD-1, HDAC	Stereotactic Radiosurgery, Nivolumab, Valproic Acid	*n* = 17	2016	I	Feasibility based on number of subjects who complete 4 doses of nivolumab; Incidence of adverse events
NCT02658279	Recurrent glioblastoma	PD-1	Pembrolizumab	*n* = 12	2016	Pilot	Response rate
NCT02311582	Malignant glioma	PD-1	Pembrolizuma, MRI-guided laser ablation, Surgical resection	*n* = 52	2015	I/II	Maximal tolerated dose (MTD) of MK-3475 when combined with MLA—Phase I only; Progression-free survival (PFS) of MK-3475 alone vs. MK-3475 plus MLA—Phase II only
NCT02337686	Recurrent glioblastoma	PD-1	Pembrolizuma, Surgical resection	*n* = 20	2015	II	Progression free survival at 6 months; Immune Effector: Treg ratio measured at the time of surgery
NCT02337491	Recurrent glioblastoma	PD-1, VEGF	Pembrolizuma, Bevacizumab	*n* = 82	2015	II	Six-month Progression Free Survival; Cohort A Recommended Phase II Dose/Maximum Tolerated Dose (MTD)
NCT02313272	Recurrent glioblastoma	PD-1, VEGF	Hypofractionated stereotactic irradiation (hfsrt), Pembrolizuma, Bevacizumab	*n* = 42	2015	II	MTD
NCT02530502	Glioblastoma	PD-1	Radiation Therapy, Temozolomide and Pembrolizumab	*n* = 50	2015	I/II	Dose-limiting toxicity and PFS (Progression Free Survival)
NCT02526017	Advanced solid tumors, including malignant glioma	CSF1R, PD-1, CD27, CSF1, IL-34	FPA008, Nivolumab	*n* = 280	2015	I	Safety, efficiency and recommended dose
NCT02423343	Advanced Refractory Solid Tumors and in Recurrent or Refractory NSCLC, Hepatocellular Carcinoma, or Glioblastoma	PD-1, TGFBR1	Galunisertib (LY2157299), Nivolumab	*n* = 100	2015	I/ II	Maximum Tolerated Dose of Galunisertib in Combination with Nivolumab
NCT02311920	Newly-diagnosed glioblastoma or gliosarcoma	PD-1, CTLA-4	Ipilimumab, Nivolumab, Temozolomide	*n* = 42	2015	I	Immune-related dose-limiting toxicities (DLTs)
NCT02336165	Glioblastoma	PD-1, VEGF	Durvalumab, Radiotherapy, Bevacizumab	*n* = 108	2015	II	Clinical Efficacy, as judged by survival, is the primary objective of the study for all cohorts but the primary endpoints differ by cohort due to the difference in patient populations
NCT01952769	Diffuse pontine gliomas	PD-1	Mdv9300 Pidilizumab	*n* = 50	2014	I/II	Testing the safety, toxicities, and efficacy of Pidilizumab
NCT02017717	Recurrent glioblastoma	CTLA-4, PD-1, VEGF	Nivolumab Nivolumab + Ipilimumab Bevacizumab	*n* = 440	2014	III	Safety and tolerability based on drug related events leading to permanent discontinuation prior to completing 4 doses; Overall Survival (OS)

## Combination administration

As tumor immunotherapy research progress, it is now recognized that combination of immune checkpoint inhibitors with conventional glioblastoma treatments such as radiation or chemotherapy may enhance the therapeutic activity. The combination therapies of immune checkpoints inhibitors with other immune checkpoints inhibitors and other kinds of cancer therapies may lead to plentiful benefits: (i) increase in cytotoxic T cells infiltration and decrease in Treg infiltration mediated by PD-1 inhibitors; (ii) preliminarily high response with comparatively low dose; (iii) potent, specific, and durable anticancer immune response compared with mono or sequential therapy (Lesterhuis et al., 2013); (iv) cytotoxic therapy may induce stress or danger signals that increase the susceptibility of tumor cells to immune modulation (Kourie and Klastersky, 2016); (v) tumor specific antigens released from dying tumor cells can activate immune system (Silk et al., 2013; Ding et al., 2014; Konstantinou et al., 2014; Vetizou et al., 2015; Hutchinson, 2016).

In the study of combination administration of different immune checkpoints inhibitors, James Larkin et al. found that combination therapy of nivolumab and ipilimumab was more effective and gained significantly longer progression-free survival compared with monotherapy, especially in patients with PD-L1—negative tumors (Larkin et al., 2015). The combination of various therapies on glioblastoma is currently explored and shows promise. A phase III trial evaluated safety and tolerability of nivolumab plus ipilimumab compared with nivolumab alone (NCT02017717).

The combination of checkpoint inhibitors with radiation also have been explored in several studies (Dovedi et al., 2014). Standard therapy of GBM consists of surgical resection followed by radiotherapy and concomitant and adjuvant chemotherapy (Stupp et al., 2005; Weller et al., 2014). Radiation exposure induces DNA damage and cell apoptosis in mature NK cells as well as T and B lymphocytes, which may inhibit the immune response (Park et al., 2014). However, the cytotoxic effect of radiation treatment helps the exposure and releasing of antigens after cell lysis thus to induce an antigen-specific response (Frey et al., 2012). Indeed, the unexpected abscopal responses in patients receiving radiation therapy during immunotherapy can promote the recruitment and function of T cells within tumor microenvironment which can complement the activity of immune checkpoint inhibitors (Pilones et al., 2015). Several clinical studies have evaluated the combination of checkpoint inhibitors with radiation. Ipilimumab combined with radiotherapy can reduce death risk in patients with melanoma brain metastases (Silk et al., 2013; Hugo et al., 2016). Indeed, emerging data using syngeneic models of multiple tumors indicated that combination of radiation and different immune checkpoint inhibitors can increase treatment efficiency. Low-doses of fractionated radiotherapy can lead to adaptive upregulation of tumor cell PD-L1 expression which is dependent on CD8+T-cell production of IFN-γ to generate effective antitumor immune response (Dovedi et al., 2014). And a phase I and II trial was initiated measuring the safety, toxicities, and efficacy of pembrolizumab in combination with MRI-guided Laser Ablation in recurrent glioblastoma (NCT02311582). Plus, another phase II trial investigating extracranial cancer such as liver, lung and melanoma with brain metastasis is now recruiting patients to test the efficacy of ipilimumab combined with stereotactic radiosurgery (NCT02107755) (Table 1).

The combination of checkpoint inhibitors with chemotherapy has also been studied for a long period (Derer et al., 2016). Ding et al. reported that combined treatment of anti-PD-1 mAb and low-dose chemotherapeutic drugs (cisplatin or paclitaxel) increased anti-cancer efficacy achieved a long-term curative effect (Ding et al., 2014). Additional studies have suggested that immune checkpoint inhibitors, combined with electrochemotherapy, enhanced antitumor benefit in advanced melanoma (Heppt et al., 2016; Hutchinson, 2016). After investigating the impact of radiotherapy (RT), chemotherapy (CT), and RCT on PD-L1 surface expression on different tumor cells, and treating with cells with different chemoradiation therapies, Derer et al. found increased PD-L1 expression in certain Melanoma and glioblastoma cells (Derer et al., 2016). Several clinical trials studied the combination of checkpoint inhibitors and temozolomide chemotherapy in GBM, which is included in the gold-standard first-line treatment of GBM (Weller et al., 2014). By producing O^6^-methylguanine in DNA, temozolomide (TMZ) mispairs with thymine, triggers futile DNA mismatch repair and causes cell death (Shao et al., 2004). GBM with an O^6^-methylguanine-DNA-methyltransferase (MGMT)-methylated promoter is associated with sensitivity to TMZ (Hegi et al., 2005). A phase II clinical trial is ongoing comparing RT + TMZ + nivolumab vs. RT + TMZ + placebo groups in patients with MGMT-methylated tumors (*N* = 320), with overall survival (OS) as the primary objective (NCT02667587). In comparison to phase II trial, a phase III clinical trial (NCT02617589) was designed to compare overall survival of nivolumab or TMZ, each in combination with RT, in newly diagnosed MGMT-unmethylated GBM patients. Recent clinical trials of checkpoint inhibitors in GBM including temozolomide were summarized in Table 1.

## Toxicity and management

Although, checkpoint immunotherapy showed remarkable success, this new strategy also lead to treatment-related toxicity. CTLA-4 and PD-1expression, actually strike a delicate balance between self-tolerance and autoimmunity (Barthel and Schatton, 2016; Kourie and Klastersky, 2016; Michot et al., 2016). Immediate toxicity of anti-CTLA-4 and anti-PD-1 antibodies is minimal; the important toxicities of these drugs are autoimmune diseases called delayed immune related side effects (irSEs) (Hamid et al., 2013; Michot et al., 2016). Immune-related side events can affect any organ systems, mainly including skin, gastrointestinal, renal, and endocrine systems (Table 2). Around 60% of ipilimumab treated patients experienced an irSEs such as rash, colitis, neuropathy, and nephritis (Michot et al., 2016). And Severe (grade 3/4) irSEs including hypophysitis, hepatitis, inflammatory colitis, epidermal necrolysis, fatal colitis, and pneumonitis can develop in 10–15% of patients in general. They occur weeks or months after treatment while side effects of chemotherapy occur rapidly, within hours or days (Michot et al., 2016). Inflammatory colitis is regarded as the most serious and life-threatening irSEs of ipilimumab, because it can advance to hematochezia, bowel perforation and peritonitis. Fortunately, many of them are asymptomatic laboratory abnormalities of unclear significance and most resolve without apparent sequelae. Recently severe atypical irSEs such as pleiomorphic manifestations or being early onset and multiple have been reported in metastatic melanoma, and a phase III clinical trial is ongoing to assess risk/benefit ratio and toxicity management of concurrent regimens (Danlos et al., 2015). In several clinical trials in GBM patients, dose-limiting toxicities and adverse events are being carefully evaluated to understand safety and tolerance of checkpoint inhibitors (Michot et al., 2016).

**Table 2:** **Summary of checkpoint inhibitor immune-related side events (irSEs)**.

**System**	**irSEs**
Gastrointestinal	Diarrhea, Colitis, Inflammatory bowel disease, Hepatitis, Increased ALT, Increased AST, and Increased bilirubin
Respiratory	Dyspnea, Pneumonitis
Renal	Renal failure, Increased serum creatinine
Skin	Pruritus, Rash, Vitiligo, Rash maculopapular, and Dermatitis
Endocrine	Hypothyroidism, Hyperthyroidism, Hypopituitarism, Hypophysitis, Adrenal insufficiency, Increased amylase, Pancreatitis, Diabetes, and Increased TSH
Neurologic	Episcleritis, Conjunctivitis, Uveitis, and orbital inflammation
Others	Fatigue, Polyarthritis, and Haematologic syndromes

Constant monitoring including regular clinical and pharmacokinetic assessments on patients is essential to prevent occurrence and deterioration of toxicity of immunomodulatory medicine. In melanoma with brain metastases, toxicity profile is measured with MRI or CT scan with contrast. Treatment requires pharmacological intervention or hospitalization (Hassel, 2016; Michot et al., 2016). Interruption of ipilimumab and the application of corticosteroids, mycophenolate mofetil, or TNF-α antagonists on the severity of the observed toxicity might be effective in the treatment. On the other hand, ongoing steroids or TNF-α inhibitor should be used with caution to treat dysimmune toxicity which can also lead to potential opportunistic infections (Michot et al., 2016).

## Discussion

Several checkpoint inhibitors have been proved potent preclinically or clinically benefits in melanoma, lung, kidney cancer, and Hodgkin's Lymphoma, and it is promising to discover new immunologic checkpoints to target GBM cells (Brahmer and Pardoll, 2013; Hamid et al., 2013; Kyi and Postow, 2014; Melero and Lasarte, 2015; Nishino et al., 2015; Robert et al., 2015; Baruch et al., 2016; Nghiem et al., 2016). Efficacy, specificity and toxicity in GBM mouse models and patients following immune checkpoint inhibitors treatment compared favorably with those in conventional anticancer therapy available in previous literatures. The high expression of checkpoint molecules in particular PD-L1 in GBM suggests that PD-L1 can be a good target for further clinical research (Jacobs et al., 2009; Vlahovic et al., 2015). Besides CTLA-4, PD-1/PD-L1, other emerging checkpoint molecules including OX40, TIM-3, and LAG3 might also be targeted effectively. Future studies will show whether combined targeting of these molecules can increase therapeutic activity. Furthermore, checkpoint inhibition targeting other immune cells such as NK cells can also help to generate better immune response to kill the tumor cells (Pegram et al., 2011).

The complex immune evasion strategies of GBM might require combination management to achieve more efficacious therapeutic benefits (Boussiotis, 2014; Chinot et al., 2014; Gedeon et al., 2014; Sakai et al., 2015; Suryadevara et al., 2015; Bordon, 2016). The optimal therapy requires a multidisciplinary approach with a thoroughly evaluating of the mechanisms of immune regulation and constant monitoring as well as pharmacological intervention to improve clinical outcomes (Ardon et al., 2012; Reardon et al., 2014; Danlos et al., 2015). Whether irSEs could be managed equally effectively with alternative immunosuppression or whether prophylactic antiviral and antibacterial therapies are beneficial in certain population need to be studied prospectively.

**Figure 2:**
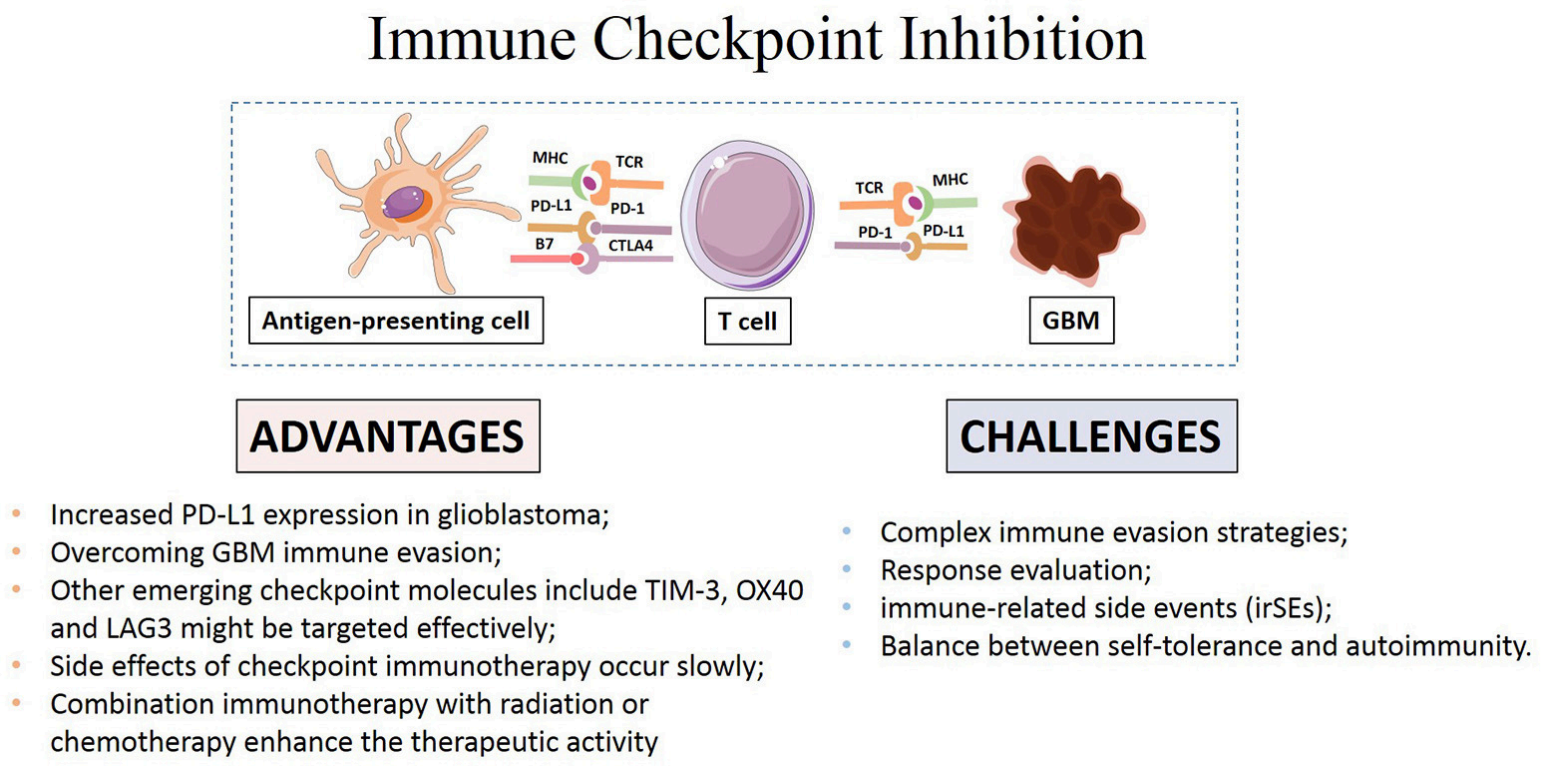
**Advantages and challenges of immune checkpoint inhibitors in GBM immunotherapy**.

How to accurately assess the response remains a main challenge in GBM immunotherapy (Eisenhauer et al., 2009; Wolchok et al., 2009). Disruption of checkpoint signaling can lead to autoimmune diseases like thyroiditis and inflammatory bowel disease (De Vries and Figdor, 2016). Thus, evaluation of immune responses to tumors and normal tissue during the application of these agents is necessary to achieve the desired anti-cancer immunity while maintaining immunologic tolerance to self-antigens expressed on normal tissue cells to avoid autoimmune response. Systematic evaluation of potential variables and local inflammation is also necessary to maximize therapeutic benefit (Bhatia and Thompson, 2014; Kopecky et al., 2014; Reardon et al., 2014; Rexer, 2015). The main advantages and challenges of checkpoint immunotherapy were summarized in Figure 2.

Despite the tremendous progress in cancer immunotherapy, the survival and other outcomes have not improved significantly in GBM therapy, which indicates deficiencies in preclinical models. Conventional mice models were challenged by the differences in mouse and human immune systems that further damage the reliable predictability. Better designed models and approaches could be one of the possible solutions for this problem. Three-dimensional organoid cultured from colorectal cancer patients showed promise in multidrug screening and personalized treatment options in colorectal cancer (Liu et al., 2016). And co-culturing intestinal epithelial organoids with intraepithelial lymphocytes (IELs) demonstrated their dynamic interaction in local immune responses (Vetizou et al., 2015). Novel biomaterials-based immune organoids with integrin ligand specificity were developed to understand the process of B cell differentiation and induction of immunological responses (Purwada et al., 2015). Moreover, newly developed human pluripotent stem cell-derived 3D organoid culture system provided a perfect *in vitro* model to study the complexity of human brain (Lancaster et al., 2013). The development of co-culture system of brain organoid with immune cells may be applied to explore the correlation of glioblastoma and immune environment and provide an effective platform for immunotherapy investigation.

## Author contributions

JH, FL, ZL, and JC conceptualized and designed the study; FL led the review process, drafted the initial manuscript, and JH reviewed all articles and extracted data; and JH, ZL, and JC analyzed and interpreted the data. All authors made substantial contributions to revising the manuscript and approved the final manuscript as submitted. JC is responsible for the overall content.

## Funding

This work was supported by National Natural Science Foundation of China (grant no. 81501163).

### Conflict of interest statement

The authors declare that the research was conducted in the absence of any commercial or financial relationships that could be construed as a potential conflict of interest.
